# Revolutionising Anatomy Education: The Current Role of Digital Technologies in Enhancing Anatomy Learning for Undergraduate Medical Students

**DOI:** 10.7759/cureus.75919

**Published:** 2024-12-18

**Authors:** Fang Fang Quek

**Affiliations:** 1 Clinical Education, Edinburgh Medical School, The University of Edinburgh, Edinburgh, GBR

**Keywords:** anatomy, digital, education, learning, technology

## Abstract

Anatomy education, which forms the cornerstone of today's medical education, has traditionally centered on cadaveric dissections and prosections as its core teaching methods. However, these methods present with challenges, including student anxiety, nausea, and limited cadaver availability. Recent advancements in digital technologies have led to the proliferation of innovative learning tools, introducing novel and transformative approaches to enhance anatomy education. While numerous studies explore the potential applications of innovative technologies in anatomy education, few studies have examined their current application in anatomy teaching for undergraduate medical students.

This gap in the literature is significant, as understanding the usability and acceptance of digital tools in anatomy teaching is crucial for ensuring that students receive high-quality education. To address this, a comprehensive review was undertaken to explore the breadth of research activity in this field, exploring the integration of digital technologies in anatomy teaching. This review aimed to address the research question: "How are digital technologies currently being used in the delivery of anatomy teaching in undergraduate medical education?". A systematic search was performed across multiple databases including PubMed, MEDLINE, Education Resources Information Center (ERIC), Embase, Scopus, and Web of Science, with studies screened using Covidence (Veritas Health Innovation, Melbourne, Australia), a web-based software platform. Eligible studies were systematically reviewed and data extracted and organised, with findings presented graphically, accompanied by descriptive narratives.

Our findings indicate that while innovative digital tools are increasingly being adopted, many medical schools continue to rely predominantly on traditional cadaver-based methods for anatomy teaching. Only a limited number of institutions have fully integrated digital technologies into their teaching practices. Additionally, traditional approaches, such as cadaveric dissections and prosections, remain the preferred choice among students for learning anatomy. A commonly reported limitation of digital tools is their difficulty in effectively conveying spatial relationships between anatomical structures, a critical component in anatomy learning. Despite this, recent studies have revealed that students and educators increasingly value multimodal approaches that combine traditional cadaver-based teaching methods with digital tools to enhance anatomy learning.

This review provides valuable insights into how digital technology is currently being utilised in anatomy teaching for undergraduate medical students. We found that while various innovative pedagogical approaches have been adopted in anatomy teaching, traditional methods such as cadaveric dissections and prosections remain the most preferred by students. While digital technology is increasingly being used to complement anatomical education, modern anatomy teaching currently adopts an integrated and multimodal approach, utilising various pedagogical methods to enhance anatomy learning. It is therefore essential that educators recognise that no single pedagogical approach suits all students and a combination of various modalities is often required to meet diverse learning needs in anatomy teaching.

## Introduction and background

The term "anatomy", derived from the Greek word "anatomē", meaning "to cut apart" or "dissect", reflects the traditional reliance on cadaveric dissections or prosections as the foundation of anatomy teaching [1]. Dissection offers a unique opportunity for haptic appreciation and enhances the conceptualisation of three-dimensional anatomical structures [2]. A survey conducted in 2007 reported that cadaveric dissections and prosections were regarded as the most effective methods for learning anatomy by students, and subsequent studies have shown that many students continue to favour these traditional approaches [3]. However, this traditional pedagogical approach is not without its challenges, as it can evoke significant student anxiety, provoke nausea, and instil a sense of mortality among students [4]. Additionally, the COVID-19 pandemic has also had an impact on body donation rates due to concerns surrounding infection risk [5]. With diminishing resources allocated for anatomy education such as time, staff, and cadaver availability, there is an increasing recognition of the pressing need to explore alternative approaches to anatomy teaching to ensure effective anatomy learning [6,7].

The global emergence of digital technologies has revolutionised pedagogy in medical education, offering novel and effective ways to enhance learning [8]. Digital learning, broadly defined as any form of education utilising technology, is increasingly recognised for its ability to deliver high-quality educational experiences, with numerous studies documenting its role in advancing both teaching and learning processes [9,10]. Digital technology is rapidly finding its way into medical education and has paved the way for transformative advancements in the teaching of anatomy. Recent technological advancements have led to the proliferation of innovative learning tools, ranging from web-based platforms and computer software to three-dimensional printing and virtual reality (VR) and augmented reality (AR), all designed to transform and enhance anatomy education [11]. These technologies enhance anatomy learning by allowing students to visualise anatomical structures in three dimensions, rather than relying on two-dimensional textbook images, facilitating a deeper understanding of the spatial relationships between anatomical structures [12].

As anatomy forms the cornerstone of today's medical education, implementing effective pedagogical methods for its teaching is essential to ensure the delivery of high-quality medical education, meeting all learning outcomes. Given the increasing integration of digital technologies into medical education, the need to explore their role in the delivery of anatomy teaching for undergraduate medical students has become imperative. A review of the literature reveals a substantial body of work documenting the potential use of innovative technologies in the delivery of anatomy teaching. While these studies are valuable, little research has been conducted to directly examine how technologies are currently being used in the delivery of anatomy teaching in undergraduate medical education. With limited research into how digital technologies are currently being used in the delivery of anatomy teaching for undergraduate medical students, little is known regarding their usability and acceptance in this aspect. The lack of such insight is a critical gap in the literature as it is especially important that all undergraduate medical learning needs are comprehensively addressed to ensure that all newly qualified doctors are safe and competent, and digital technology is one important educational method of achieving this [13]. Therefore, this paper seeks to address the ubiquitous research question: "How are digital technologies currently being used in the delivery of anatomy teaching in undergraduate medical education?".

## Review

Methodology

This review aims to address the research question: "How are digital technologies currently being used in the delivery of anatomy teaching in undergraduate medical education?".While there are numerous studies exploring the potential applications of innovative technologies in anatomy education, few directly examine their current use in undergraduate medical education. To address this research gap, a review was undertaken to explore the breadth of research activity in this field, identify research gaps, and synthesise findings for dissemination. 

Ethical consideration

As this study involves working only with secondary data and does not involve direct interaction with human participants, formal ethical approval from ethics committees such as the Health Research Authority (HRA) is not required. In accordance with the British Educational Research Association (BERA) Ethical Guidelines, the author declares no conflict of interest and confirms that no funding was received for this study.

Research design

This review was conducted in accordance with the Joanna Briggs Institute (JBI) methodology for scoping reviews, comprising nine structured stages to ensure a rigorous and systematic approach.

Identifying the Research Question

Anatomy, regarded as the cornerstone of surgical education, remains a foundational subject in today's modern surgical practice [14,15]. The importance of surgical education has been underscored by initiatives such as the "No Training Today, No Surgeons Tomorrow" campaign led by the UK Joint Committee for Surgical Training (JCST) [16]. While there are numerous studies in the literature documenting the potential of innovative technology in anatomy education, studies specifically examining the current use of these technologies are few and far between. The lack of such insight is a critical gap in the literature. To address this critical gap, the research question for this study was identified: "How are digital technologies currently being used in the delivery of anatomy teaching in undergraduate medical education?".

Developing Eligibility Criteria

To ensure the review's findings were directly aligned with its objectives, relevant studies were assessed against predefined eligibility criteria.

Inclusion/exclusion criteria: This review exclusively includes studies focusing on the use of digital technologies in the delivery of anatomy teaching in undergraduate medical education, focusing specifically on undergraduate medical students. Studies involving other disciplines, such as nursing, dentistry, or other allied health professions, were excluded. This criterion ensures that the findings address the unique educational needs of undergraduate medical students in anatomy learning. By narrowing the scope to this population, this review provides targeted insights into the integration of digital technologies in anatomy teaching within the broader framework of undergraduate surgical education. All studies involving the use of digital technologies in the teaching of anatomy in undergraduate medical settings were included in this review. Studies were included if they assess the use of digital technology in the delivery of anatomy teaching within undergraduate medical education settings. Studies involving postgraduate medical and/or surgical trainees, defined as individuals who had already completed their training and qualified as a doctor, were excluded. Additionally, only studies published in the English language were considered in this study, due to constraints on translation resources.

Search Strategy

A comprehensive search strategy was conducted using the search terms "technology" OR "simulation" OR "virtual reality" OR "augmented reality" OR "digital" OR "online" AND "anatomy" AND "undergraduate" OR "surgical" on multiple electronic bibliographic databases including PubMed, MEDLINE, Education Resources Information Center (ERIC), Embase, Scopus, and Web of Science. During the preliminary search for studies pertinent to this research question, terms and phrases extracted from the titles, abstracts, and indices of relevant papers guided the formulation of the search strategy. To broaden the scope of the search, grey literature was also utilised using internet search tools such as Google Scholar to identify informative sources. These terms were then refined, and all relevant search terms were identified to readjust the search strategy. To ensure that all relevant records would be retrieved regardless of the terminologies used by the author, searches were executed using both free-text and Medical Subject Headings (MeSH) terms. The search terms were also combined using Boolean operators to ensure all relevant citations were captured. To identify all pertinent studies on this subject and to explore if there was any change in the trend of technology use in the delivery of undergraduate surgical teaching, a start date for the search was not imposed.

Study Selection

All potentially relevant studies identified through the search strategy were assessed for inclusion based on predefined eligibility criteria using a systematic two-stage process. In the first stage, titles and abstracts of all retrieved studies were screened to identify those potentially meeting the criteria. In the second stage, full texts of studies deemed eligible were reviewed in detail to confirm their inclusion, as per the pre-established criteria. To enhance the efficiency and accuracy of this process, the web-based software platform Covidence (Veritas Health Innovation, Melbourne, Australia) was employed to streamline study screening and management.

**Figure 1:**
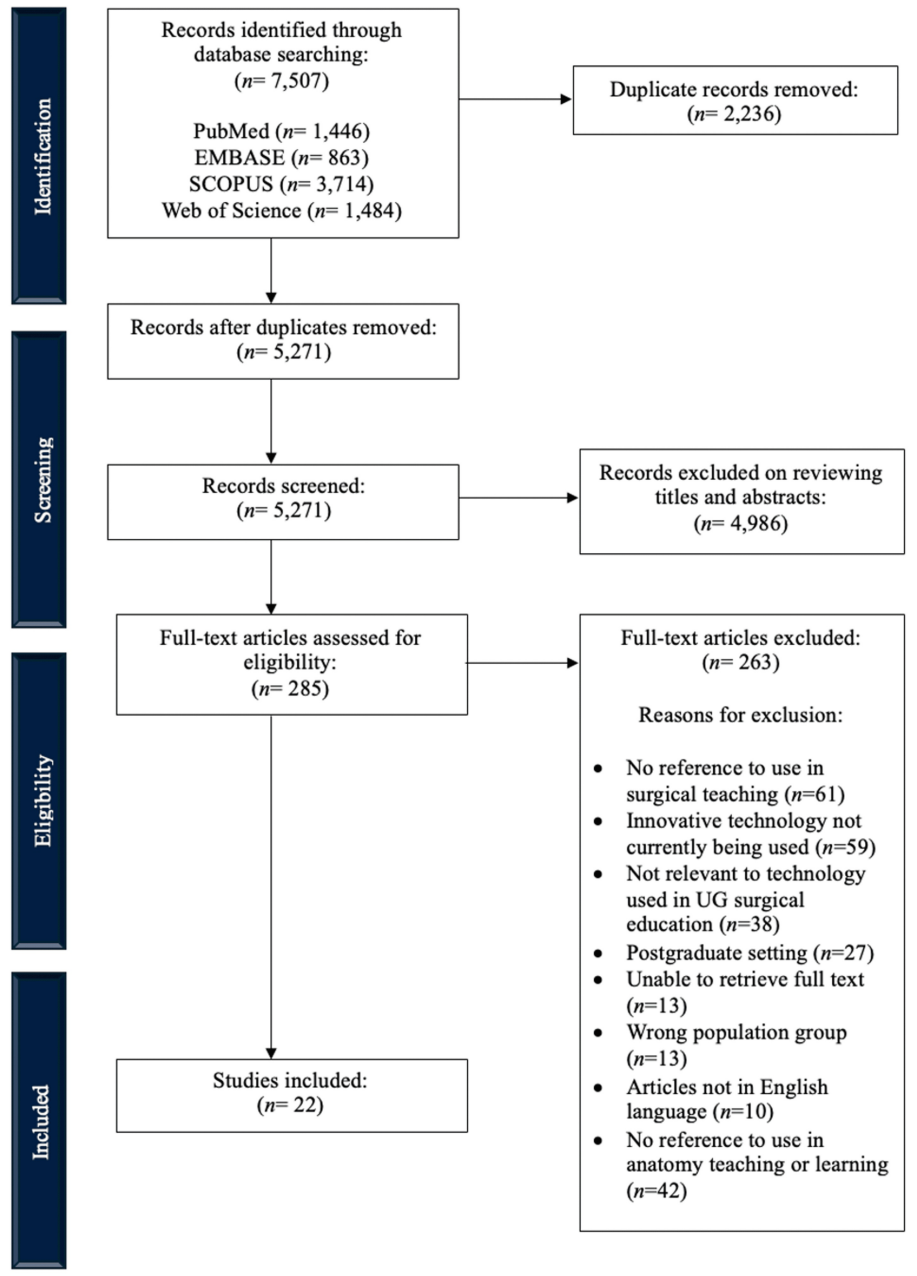
PRISMA-ScR flow diagram for the study selection process. PRISMA-ScR: Preferred Reporting Items for Systematic Reviews and Meta-Analyses extension for Scoping Reviews

The initial search resulted in 7,507 studies. Once duplicates were removed, 5,271 studies were assessed for eligibility, and 4,986 were excluded after the screening of the abstract to evaluate if the article warranted full-text review based on the predefined inclusion and exclusion criteria. After the full-text review, 22 studies met the eligibility criteria and were included in the final analysis. Figure 1 illustrates the schematic diagram of this study's methodology.

Extracting the Evidence

All included studies were meticulously reviewed, and data were systematically extracted using a standardised data extraction form created in Microsoft Excel (Version 16.91, 2024) (Microsoft Corp., Redmond, WA, USA). A detailed charting form was developed to capture all essential characteristics and key information from each study, ensuring consistency and comprehensiveness. To optimise its effectiveness, the data extraction form was pilot-tested and refined based on insights gained during the initial trial phase. This iterative process ensured the reliability and accuracy of the data collection framework, facilitating a thorough synthesis of findings across all included studies.

Analysis of the Evidence

All collected data were systematically organised and analysed to facilitate graphical representation alongside a description narrative. A thematic analysis was subsequently conducted to identify, analyse, and report common patterns and themes across the included studies. The thematic review was performed in three iterative stages: during study selection, at the data extraction stage, and during the final review once all data had been charted. As part of this iterative process, codes were reviewed and refined at each stage, with additional themes added as they emerged throughout the process. Through repeated and active engagement with the included studies, including thorough reading and familiarisation with the data, detailed notes were taken on potential codes and relationships between concepts, providing a foundation for subsequent theme development. Recurring concepts and findings were then systematically coded and organised into overarching themes.

Results

Types of Publications

**Figure 2:**
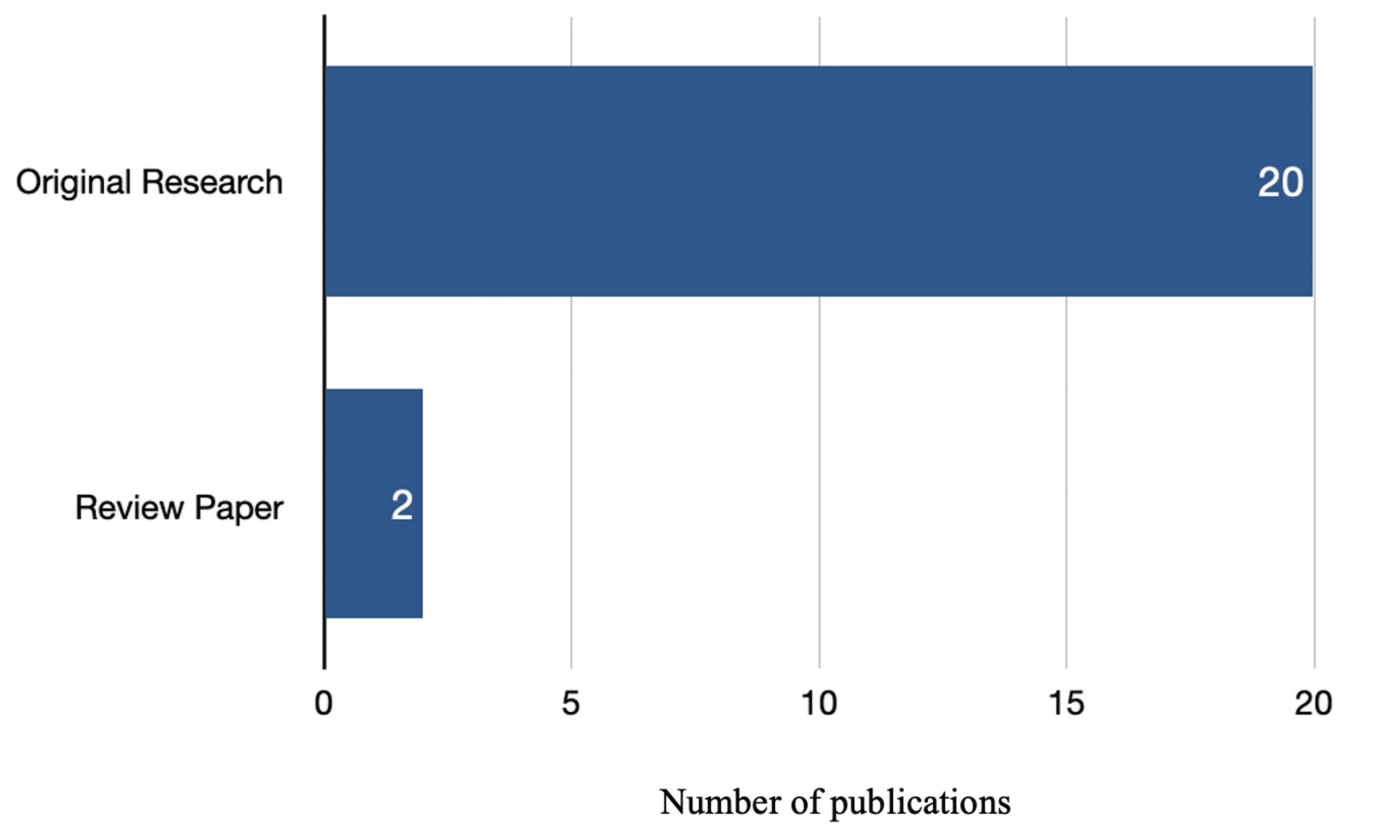
Types of publications included in the study.

Among the 22 studies included in our analysis, 21 were published as full papers, while one was available only as an abstract. As shown in Figure 2, the majority of these studies (n=20) were original research, and two were review papers.

Year of Publications

**Figure 3:**
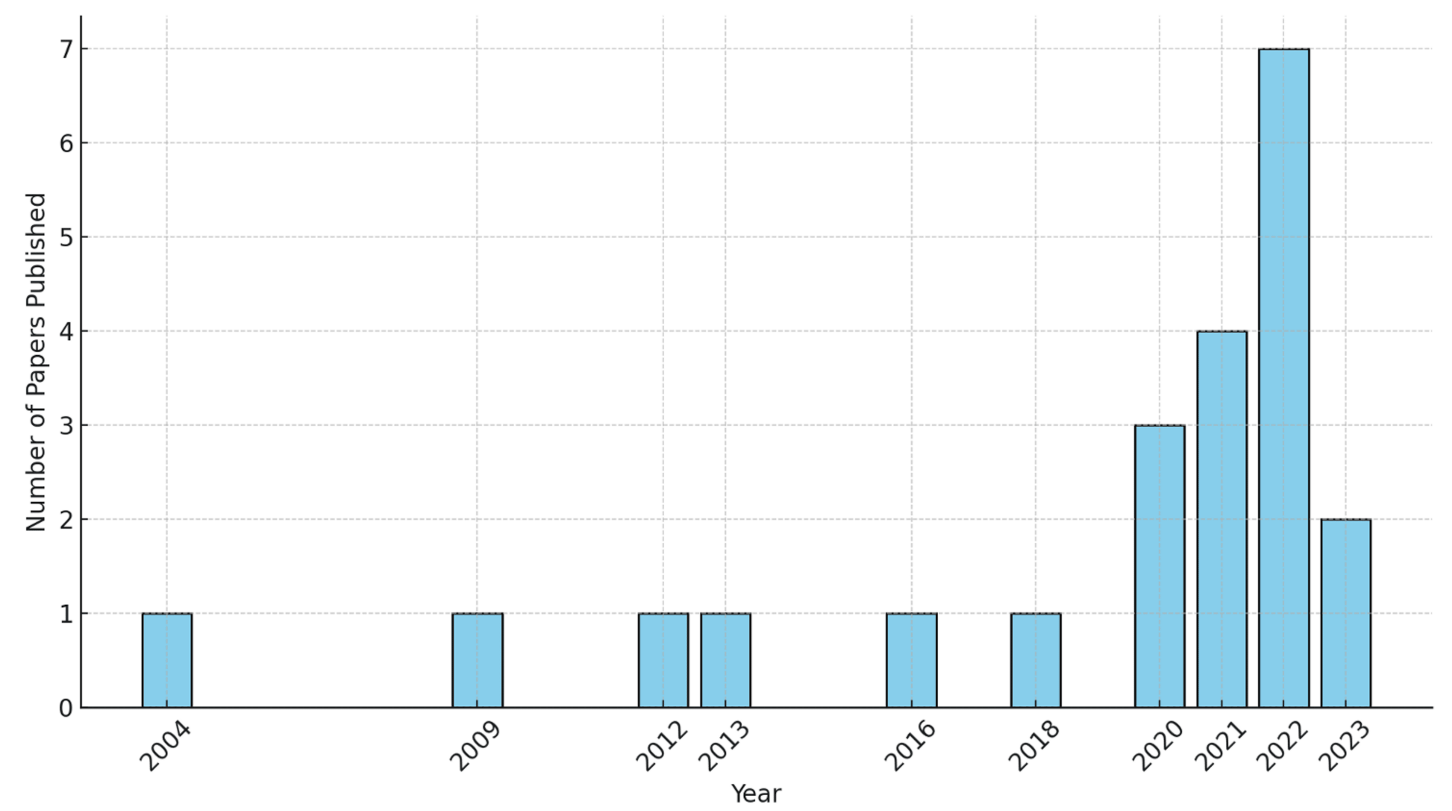
Number of papers published each year.

To assess the trend in the utilisation of digital technology in the delivery of anatomy teaching within undergraduate medical education, a specific date range was not imposed for this study. Among the included articles, the earliest study was published in 2004. As shown in Figure 3, the included studies reveal a gradual increase in the number of publications over the years, with a notable surge in recent years, particularly from 2020, suggesting a growing interest and increasing research activity in this field, likely related to the emergence of the COVID-19 pandemic.

Geographical Locations of Studies

**Figure 4:**
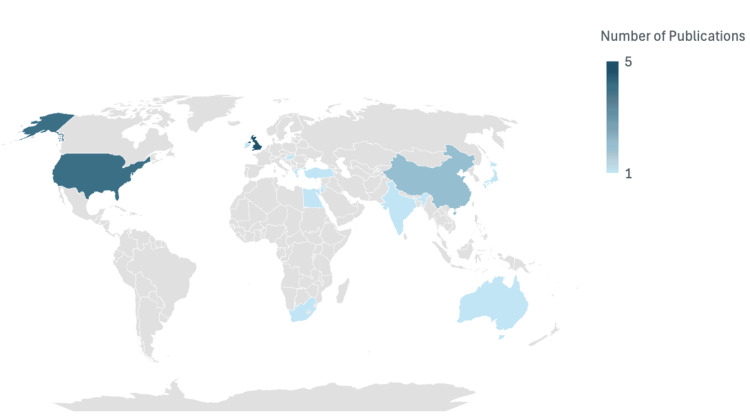
Geographical distribution of studies included in the review.

As shown in Figure 4, most studies included in this review were conducted in the United Kingdom (five out of 22 studies), followed by the United States. The rest of the studies were spread across different countries as shown in Figure 4. Overall, this review included studies produced across 13 different countries.

Types of Digital Technology Currently Used in Anatomy Teaching in Undergraduate Medical Education

**Figure 5:**
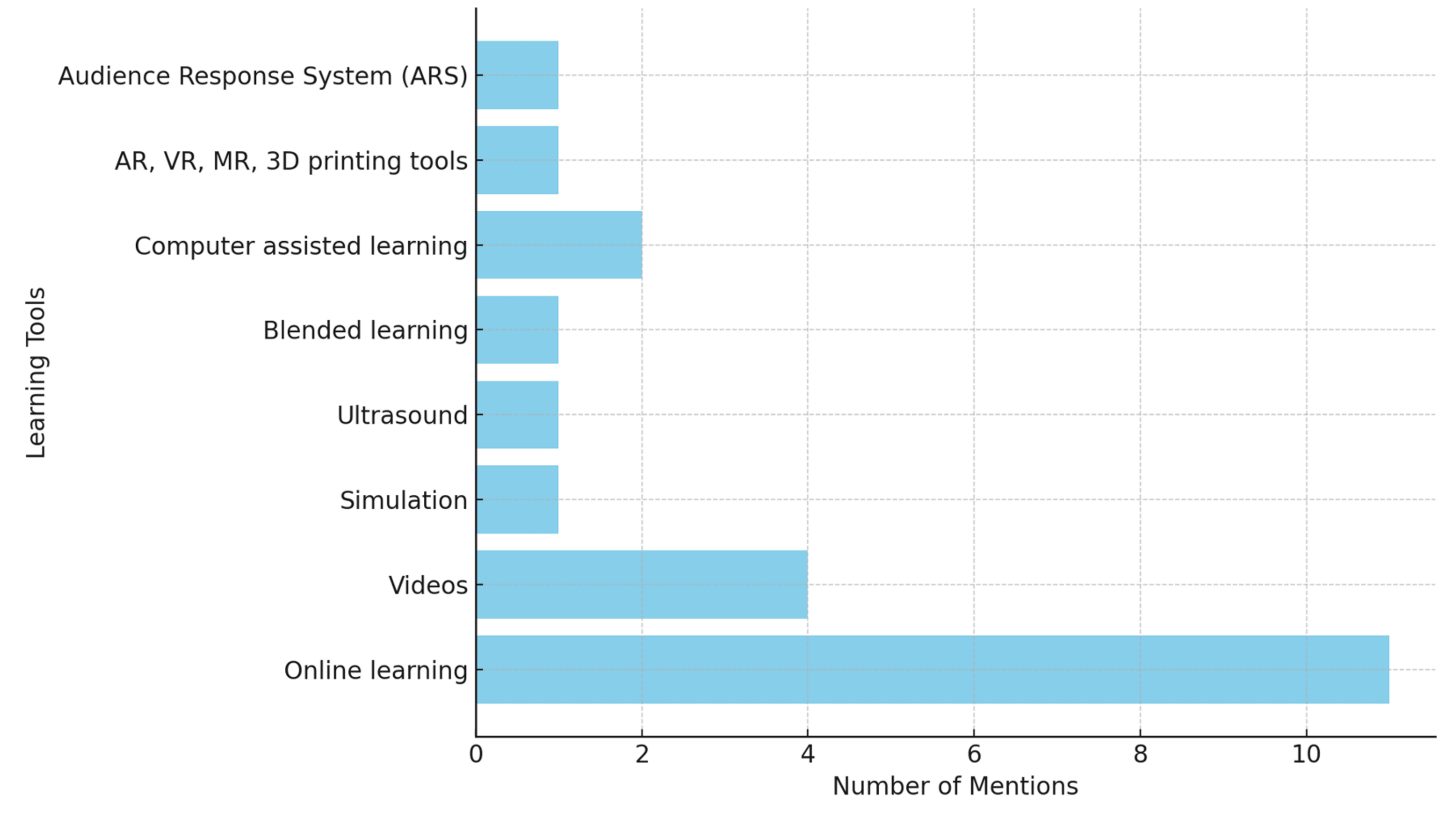
Types of technology used in the delivery of anatomy teaching for undergraduate medical students. AR: augmented reality; VR: virtual reality; MR: mixed reality

Figure 5 illustrates the various types of technology used in the delivery of anatomy teaching for undergraduate medical students. Among the 22 studies analysed, online learning emerged as the most prevalent pedagogical approach. The remaining studies reported the utilisation of technology across different modalities including audience response system (ARS), simulation, computer-assisted learning, multimedia tools such as dissection videos, flipped classrooms, and advanced technologies like VR and AR. Although there were numerous studies in the literature reporting on the potential use of innovative technologies such as VR, AR, mixed reality (MR), and 3D printing tools in the delivery of anatomy teaching, only one study reported the current usage of these advanced technologies in this area. 

Thematic Analysis

**Figure 6:**
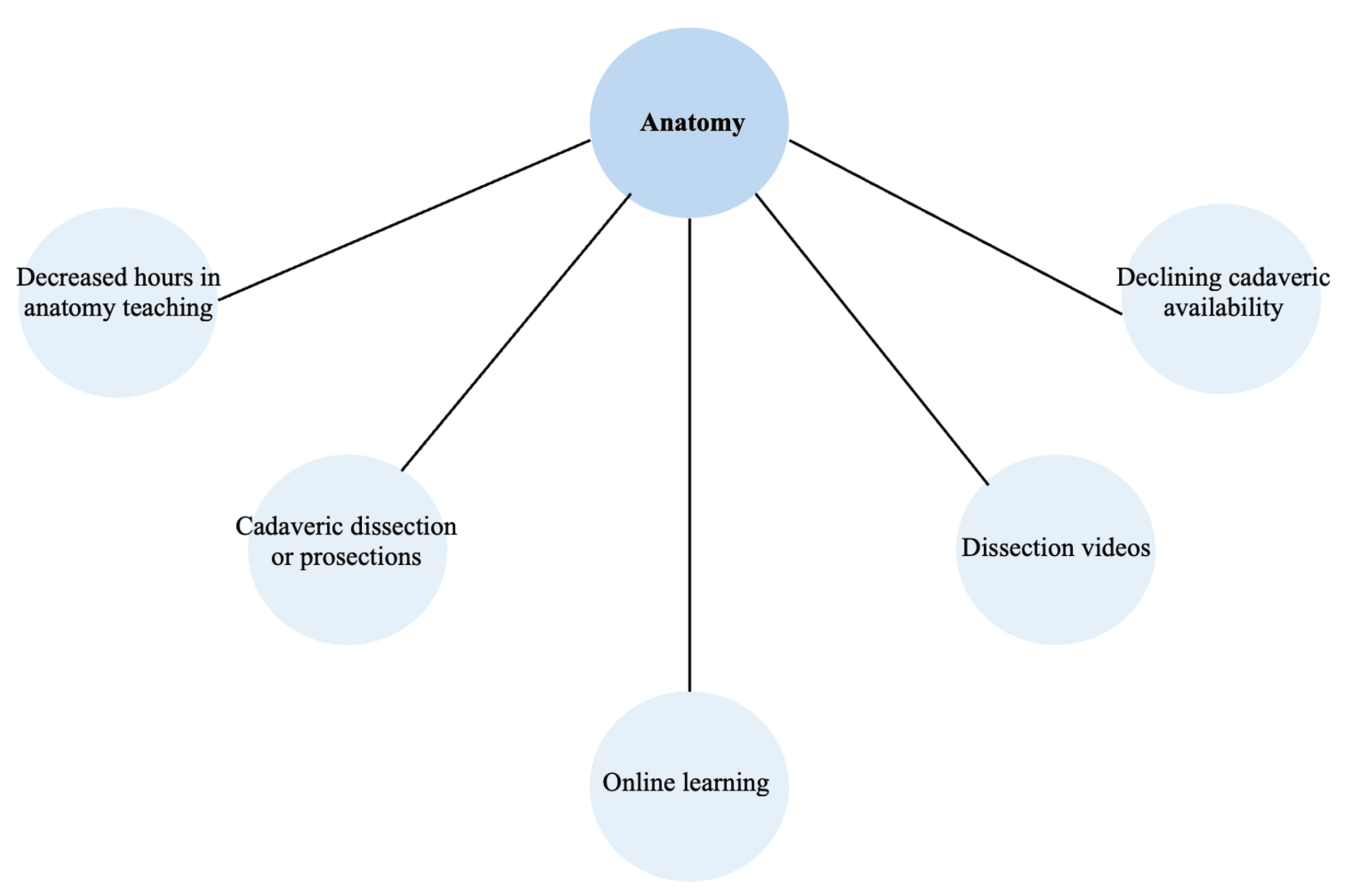
Diagrammatic representation of the thematic analysis tree.

Codes identified from the thematic analysis were subsequently grouped into overarching themes relating to the utilisation of digital technology in delivering anatomy education for undergraduate medical students. Figure 6 is a diagrammatic representation of the thematic analysis tree.

Discussion

Anatomy in Undergraduate Surgical Education

Anatomy is a subject characterised by its high intrinsic cognitive load, requiring learners to retain extensive information, master complex terminology, and develop spatial awareness to understand intricate anatomical relationships [17]. As the cornerstone of medical education, its significance in clinical practice is universally acknowledged. However, despite its critical importance, anatomy has been increasingly marginalised within the medical curriculum, primarily as a result of ongoing educational reforms. The rapid expansion of medical knowledge has driven initiatives such as the Tomorrow's Doctors of the General Medical Council (GMC) to address the challenges of information overload faced by medical students [18]. While these reforms aim to streamline learning, they have inadvertently diminished the emphasis on anatomy within the curriculum. This has raised significant concerns about inadequate foundational knowledge among medical graduates, with evidence linking poor anatomical understanding to an increase in medical errors [14,19]. These deficiencies are particularly evident in surgical practice, where a growing number of litigation cases have been attributed to deficiencies in anatomical knowledge [14]. Notably, the most common cause of surgical litigation claims in the United Kingdom involves damage to underlying structures during procedures, further emphasising the consequences of insufficient anatomy training [20].

Such findings highlight the critical importance of a robust anatomical education, a necessity consistently emphasised in the literature. However, diminishing resources for anatomy education, including reduced teaching hours, limited availability of cadavers, and fewer dedicated staff, pose substantial challenges. This has led to an increasing recognition of the need to explore alternative and innovative approaches to anatomy teaching, ensuring medical students acquire the comprehensive anatomical knowledge essential for safe and effective clinical practice. This study sought to explore the existing literature on the use of digital technologies in the delivery of anatomy education to undergraduate medical students. A comprehensive review was conducted, analysing 22 studies that met the inclusion criteria.

Digital Technology Used in the Delivery of Anatomy Teaching

The most commonly reported digital tools used in anatomy teaching include computer-assisted learning platforms and online learning resources. Dissection, an essential component of anatomy learning, is particularly demanding for students due to its inherent complexity and steep learning curve. These challenges are further compounded by the limitation in resources including the availability of cadavers and instructors, highlighting the need for innovative approaches to support and enhance traditional anatomy teaching methods [21]. Numerous studies have highlighted the effectiveness of multimedia tools like digital atlases, web-streamed lectures, and instructional videos in supporting anatomy education [22]. A study by DiLullo et al. highlighted the various ways in which students utilise dissection videos to enhance their anatomy learning, including exam preparation and lab dissection practice [23]. Another study also highlighted the effectiveness of dissection videos in helping students grasp anatomical concepts and visualise anatomical relations [7]. Cadaveric videos created by instructors have been well-received by students as valuable resources for supplementing their anatomy learning. These tools, such as virtual dissections and animations, have gained prominence for their ability to support self-paced learning and provide interactive experiences, which have been shown to enhance student motivation [24].

In addition, anatomy applications have also emerged as an invaluable tool in anatomy education, providing access to high-quality anatomical materials, enabling students to study and review content at their convenience, particularly when laboratory access is unavailable [25]. In a study by Dua et al. evaluating the effectiveness of a peer-sharing application used to supplement anatomy learning where students share images of anatomical structures from their cadaveric dissections in a secure platform, students reported that they found this useful in solidifying their knowledge and for self-assessment in preparation for practical examinations [25]. Besides, this application also promotes collaborative learning where students are able to explore anatomical variations across different specimens and students reported that they found the ability to zoom within an image and the annotation function on the images helpful [25]. These digital tools make them a useful adjunct in anatomy teaching, addressing challenges posed by the limited resources in anatomy education.

A review by Adnan and Xiao reported the use of advanced technologies in anatomy education at certain institutions [26]. VR, a technology which uses software to create immersive experiences, enables users to feel as if they are in a different environment, offering a platform for delivering learning experiences which may be challenging or impossible to replicate in real-life settings [27]. Studies have reported that VR enhances anatomy learning by allowing students to visualise anatomical structures in three dimensions rather than relying on two-dimensional textbook images, facilitating a deeper understanding of the spatial relationships between anatomical structures [12]. Recent advancements in technology, particularly the development of modern head-mounted displays like Google Glass (Google, Mountain View, CA, USA), Samsung Gear VR (Samsung Electronics, Suwon, South Korea), and Microsoft HoloLens (Microsoft Corp., Redmond, WA, USA), have significantly expanded the applications of VR, particularly in the field of medical education [28]. AR, a form of MR, is also an emerging technology which superimposes digitally generated 3D representations onto real-world environments. Unlike VR, which immerses users in a completely synthetic environment which separates them from their actual surroundings, AR allows users to engage with their real environment in real time while interacting with virtual elements [29]. Numerous studies have also reported the potential utilisation of AR in medical education, particularly in anatomy learning, where it allows students to interact with digital anatomical representation from all angles, offering immersive experiences which aid understanding [26]. Interestingly, although there were numerous studies in the literature reporting on the potential use of innovative technologies such as VR, AR, MR, and 3D printing tools in anatomy education, only one study reported the current usage of these advanced technologies in the delivery of anatomy education for undergraduate medical students.

One interesting finding from this review is that despite advancements in educational technology and all the positive comments about digital tools in anatomy teaching, many medical schools continue to rely predominantly on traditional methods for teaching anatomy such as cadaveric dissections and prosections, with only a few leading institutions fully integrating technological tools for the delivery of anatomy teaching [30]. As reported by several studies included in this review, although many students found online learning helpful in supplementing anatomy learning, the majority of students still felt that traditional cadaveric teaching cannot be replaced [31,32]. The most commonly cited reason for this is due to the difficulty in appreciating the spatial orientation and visualisation of relationships between anatomical structures using virtual learning [33]. Cadaveric teaching, on the other hand, allows for the visualisation of anatomical detail and appreciation of anatomical variation which is essential in the learning of anatomy [34]. The use of cadaveric dissections or prosections still remains the "gold standard" in today's anatomy teaching [34,35]. Research indicates that medical students most highly value a combination of cadaveric prosections and active learning tutorials with supplementary dissection videos and electronic resources [7]. In practice, modern anatomy teaching adopts an integrated and multimodal approach, utilising various pedagogical methods to enhance anatomy teaching and learning [36].

## Conclusions

The key findings from this review provide valuable insights into how digital technology is currently being utilised in anatomy teaching for undergraduate medical students. By exploring the breadth of research in this area, this review has summarised and discussed key findings identified from the analysis of all included studies. We found that while various innovative pedagogical approaches have been adopted in anatomy teaching, traditional methods such as cadaveric dissections and prosections remain the most preferred by students. However, studies have also shown that digital technology is increasingly being used to complement anatomical education. In practice, modern anatomy teaching adopts an integrated and multimodal approach, utilising various pedagogical methods to enhance anatomy teaching and learning. It is therefore essential that educators recognise that no single pedagogical approach suits all students and a combination of various modalities is often required to meet diverse learning needs in anatomy teaching.
